# Profile of Patients With Obstructive Sleep Apnea: An Initial Experience in a Tertiary Health Facility

**DOI:** 10.7759/cureus.64169

**Published:** 2024-07-09

**Authors:** Jane S Afriyie-Mensah, Robert Aryee, George Aryee, Ernest Amaning-Kwarteng, Osei Kankam

**Affiliations:** 1 Internal Medicine, University of Ghana Medical School, Accra, GHA; 2 Cardiology, University of Ghana Medical Centre, Accra, GHA; 3 Anesthesia, Korle-Bu Teaching Hospital, Accra, GHA; 4 Respiratory Medicine, Conquest Hospital, East Sussex Healthcare NHS Trust, St. Leonards-on-Sea, GBR

**Keywords:** stop-bang scores, obesity, obstructive sleep apnoea (osa), oxygen desaturation index (odi), apnoea-hypopnoea index (ahi)

## Abstract

Introduction

Obstructive sleep apnea (OSA) is the most common sleep-related breathing disorder with increasing prevalence worldwide. The disease is, however, underdiagnosed in many resource-limited countries, especially in sub-Saharan Africa with unknown prevalence.

Study aim

The aim of this study was to determine the demographic and clinical characteristics, as well as measured sleep study parameters of suspected OSA patients.

Methods

The study was a retrospective review of the clinical characteristics and home sleep study reports of patients seen at the respiratory and sleep clinic from January 2020 to June 2022. Descriptive statistics such as means, medians, and percentages were employed to summarize the data using tables and graphs. Spearman correlation coefficient and Fisher’s exact test were used to determine associations between the variables.

Findings

The study participants were predominantly male, and 64.7% were ≥50 years of age. Approximately 76% of the cases had moderate-to-severe OSA based on the apnea-hypopnea index (AHI) scores with a mean BMI of 38.4kg/m^2^ and 43.1kg/m^2^, respectively (p=0.013), and a mean STOP-BANG score of 5.2 and 6.2, respectively (p <0.001). There was a positive correlation between AHI scores and BMI of the patients (r=0.252, p=0.003), as well as with their STOP-BANG scores (*r*=0.436, p< 0.001). Oxygen desaturation index (ODI) parameters of participants also positively correlated with the AHI scores (*r*=0.872, p<0.001).

Conclusion

The proportion of patients with moderate-to-severe OSA was high. Obesity was significantly associated with AHI scores, which also positively correlated with the STOP-BANG and ODI scores. These results suggest that the burden of OSA, which is closely linked with obesity, could be underestimated in Ghana and requires epidemiological studies in the very near future to clearly define and anticipate its impact on the health economy of Ghana.

## Introduction

Obstructive sleep apnea (OSA) is the most prevalent sleep-related breathing disorder characterized by recurrent sleep-dependent breathing pauses and reductions in airflow with associated hypoxia and frequent arousals [1]. The disease is commonly seen worldwide in both developed and developing countries but is highly under-reported in the latter [2]. Although prevalence is variable across countries, global estimates suggest that around a billion people worldwide have OSA [2]. In the United States, around 25-30% of men and 9-17% of women meet the criteria for OSA [3]. In Europe, Malhotra et al. estimated that around 90 million people had moderate-to-severe OSA [4]. Estimates from most developing countries including Africa are scanty, with few qualitative surveys employing sleep apnea predictive tools to identify patients at an increased risk of having OSA [5-7]. This comparative gap in data can be largely attributed to the lack of disease awareness in most developing countries and limited skill set (sleep/respiratory physicians) and resources to diagnose and manage OSA. Additionally, the general disinterest by policymakers in resource-constrained settings with limited supportive health insurance schemes is a key negative factor, bearing in mind the cost implications of diagnosing and managing OSA [8].

The global increase in OSA prevalence is closely linked to increasing obesity rates as well as the availability of improved diagnostic tools in most countries [9]. In the Wisconsin longitudinal analysis, researchers reported that a 10% increase in body weight resulted in a six-fold increased risk of OSA [10]. The disease prevalence has been shown to be higher in males and with increasing age, particularly in persons older than 60 years [11]. Racial disparities of OSA have been observed in population-based studies, in which African Americans, Hispanics, and Asians were found to be at a higher risk of developing OSA [12,13]. The underlying mechanism of OSA is pharyngeal airway narrowing or closure during sleep with multiple structural and non-structural etiological risk factors at play [14]. Current knowledge has shown that the role played by mechanical load on the pharyngeal airways by excess adipose tissue as in obesity is key in the development of OSA [14]. Other structural defects such as retrognathia, micrognathia, tonsillar/adenoid hypertrophy, and other specific craniofacial variations in genetic abnormalities such as Down’s syndrome and Prader-Willi syndrome also cause appreciable airway narrowing during sleep [15]. Hypothyroidism and acromegaly are associated with crowding of the upper airway through increased subcutaneous tissue deposition. Impaired pharyngeal muscle tones during sleep, a low threshold for arousal in response to airway narrowing, and unstable control of breathing in sleep are equally significant non-anatomical causes of OSA [14]. Alcohol abuse, smoking, and use of sedatives or hypnotics are known to accentuate airway narrowing in at-risk individuals.

The characteristic complete or partial upper airway obstruction during sleep can cause significant physiologic disturbances, with adverse health implications in affected individuals, and has been associated with increased morbidity and mortality [16,17]. Unmanaged OSA negatively impacts multiple organ systems, particularly the metabolic, cardiovascular, and cerebrovascular systems, leading to frequent and longer hospitalizations, poor disease outcomes, and higher healthcare costs [18].

Locally, knowledge of OSA as a disease entity has been very low due to the lack of awareness among clinicians and lack of sleep apnea diagnostic tools to aid objective diagnosis. Similarly, there has been very limited research in sleep apnea in our setting with reference to just one qualitative OSA risk assessment among stroke patients using the STOP-BANG predictive tool [7]. The recent availability of a portable home sleep apnea test (HSAT) device in our facility has not only led to an increased disease awareness and referral of suspected cases for testing but also has offered the unit a great opportunity to objectively determine the baseline clinical characteristics of patients with OSA and quantitatively evaluate associations between the measured sleep test parameters such as the apnea-hypopnea index (AHI), oxygen desaturation index (ODI), and pre-test probability scores with BMI as well as existing comorbidities in the diagnosed patients. This study is novel in our setting and therefore seeks to provide a platform for future epidemiological and clinical studies in OSA needed to define the burden of disease and guide enactment of local management policies which are currently non-existent.

## Materials and methods

Study design

This was a hospital-based retrospective review of the sleep study reports of patients who underwent a home sleep test at the respiratory unit between January 2020 and June 2022.

Study site

The study was conducted at the Physiology Lab of the Department of Medicine and Therapeutics of the Korle-Bu Teaching Hospital, a premier tertiary facility that serves as a major local and regional referral center in West Africa. The department houses a physiology unit where other investigations such as pulmonary function tests, 6 minutes’ walk test (6MWT), and electroencephalogram (EEG) are conducted.

Study population

All sleep records of adult patients, aged 18 years and above, were eligible for the study. Incomplete sleep report forms with missing data were excluded from the study.

Sleep testing

The sleep device used was Alice NightOne (Philips Respironics, Murrysville, PA, USA). This is a portable type III sleep device appropriate for home sleep testing. It has an effort belt, a nasal cannula, and an oximeter. The basic channel set of the device gives information on a body positions during sleep, SpO_2_ and pulse rate through the pulse oximeter, respiratory effort through the effort belt, snoring, oro-nasal airflow, and flow pressure through the cannula. The sensors in the device fit recommendation by the American Academy of Sleep Medicine (AASM). Patients' demographic data (age, gender, and BMI), smoking and alcohol history, comorbidity present (hypertension, history of stroke, diabetes, hyperlipidemia, and ischemic cardiac events), and STOP-BANG scores are routinely captured by the lab onto a sleep report form prior to testing. The STOP-BANG questionnaire is a validated tool used to assess patient’s risk of having OSA and involves questions on snoring, tiredness, observed apnea, blood pressure, BMI > 35kg/m^2^, age > 50 years, neck circumference > 40cm, and male gender, which are scored [19]. Each parameter when present attracts a score of 1; a total score of ≥3 implies a likelihood of having OSA, while a score of ≥5 predicts a moderate-to-severe risk of OSA. Prior to performing the sleep test, the purpose of the procedure and process of fixing the portable device was fully explained to patients through a detailed demonstration and, in some cases, video recording. Upon return, the recorded information is downloaded onto a monitor for viewing and generation of a detailed sleep report. Parameters recorded by the sleep device included duration of monitoring, oxygen desaturation (drops in pulse oximetry values of 4% or more), hypoxemic burden (proportion of time spent in oxygen-desaturated states, where patients’ pulse oximetry measures are below <90%, <85% or 75% during sleep), AHI, ODI, percentage duration of snoring during sleep, and the heart rate. Tracing of waveforms of nasal flow, thoracic movements, sleep positions assumed, breathing pattern, and oxygen desaturation are also viewed on the monitor for interpretation.

OSA was diagnosed using AHI scores according to the American Association of Sleep Medicine [20]:

Mild OSA: An AHI of at least five events/hour but fewer than 15 events/hour

Moderate OSA: An AHI of at least 15 events/hour to 30 events/hour

Severe OSA: An AHI of greater than 30 events/hour

For the purposes of the study, patients with AHI ≥ 15 were considered as having significant OSA. Scoring of ODI was as according to the American Association of Sleep Medicine [20]:

Mild: ODI of at least five events/hour, but fewer than15 events/hour

Moderate: ODI I of at least 15 events/hour to 30 events/hour

Severe: An ODI of greater than 30 events/hour

Data collection

Convenient sampling was employed, where all eligible sleep reports over the specified period were included in the study. Reports with incomplete demographic data and measured sleep parameters were excluded by the team of research assistants. Sleep reports were assigned unique codes to conceal patients' identity and relevant data extracted using an abstraction tool. Information extracted included patients' age, gender, BMI, STOP-BANG scores, comorbidities, smoking status, AHI, and ODI values. Data extraction was done by two research assistants independently and compared to avoid extraction error. Where comorbidities were neither ticked as yes or no, it was deemed to be non-existent. Data were entered into Microsoft Excel 2016 (Microsoft Corp., Redmond, WA), which was password-protected.

Data analysis

Data were cleaned and analyzed using SPSS Version 25 (IBM Corp., Armonk, NY). Continuous variables such as age, BMI, and pre-test probability scores were summarized as means (±SD) or median (interquartile ranges) where appropriate. Categorical variables such as gender, occupation, and comorbidities were summarized as frequencies and proportions. Fisher’s exact test was used to determine the association between categorical variables. Spearman correlation coefficient was used to determine the associations between AHI/ODI and BMI and pre-test probability scores. A p-value of less than 0.05 was considered statistically significant.

Ethical considerations

Ethical approval was obtained from the Institutional Review Board of the Korle-Bu Teaching Hospital (STC/IRB/000109/2023).

## Results

A total of 144 eligible sleep study records were obtained, with majority (60.4%) being males. The mean age was 54.4 (±14.8) years, with 67.4% aged 50 years or older. Mean BMI was 40.6 (±10.4) kg/m^2^, and a significant proportion (94.4%) were non-smokers. Comorbidities were present in nearly 48% of the cases. Around 25% had hyperlipidemia, 12% had hypertension, and 4.9% with diabetes mellitus (Table 1).

**Table 1:** Demographic and health characteristics

Variable	Descriptive
Sex, n (%)	
Male	87 (60.4)
Female	57 (39.6)
Age (years), mean (± SD)	54.4 (±14.8)
<20	2 (1.4)
20-29	7 (4.9)
30-39	16 (11.1)
40-49	22 (15.3)
50-59	41 (28.5)
≥60	56 (38.9)
BMI (kg/m^2^), mean (±SD)	40.6 (±10.4)
Smoking status, n (%)	6 (5.6)
Hypertension, n (%)	17 (11.8)
Diabetes mellitus, n (%)	7 (4.9)
Heart failure, n (%)	3 (2.0)
Hyperlipidemia, n (%)	36 (25.0)
Thyroid disease, n (%)	4 (2.8)
Ischemic heart disease, n (%)	2 (1.4)

Around 72% (104) of all who underwent sleep study had significant OSA per study definition (AHI ≥ 15 events/hour) and more than half of these had AHI > 30 (severe OSA). Those with a negative sleep test (AHI < 5) were 7 (0.05%), and those with insignificant OSA (AHI < 15 events/hour) were around 23% (33). Almost a quarter (74%) had a STOP-BANG score of ≥5 (high risk), while 24% scored between 0 and 2 (low risk) (Table 2).

**Table 2:** AHI categorization and STOP-BANG score among patients AHI, apnea-hypopnea index

Variable	n (%)
AHI severity	
15-30 (moderate)	31 (22.6)
>30 (severe)	73 (53.3)
STOP-BANG scores	
Low risk (0-2)	35 (24.3)
Intermediate risk (3-4)	3 (2.1)
High risk (≥5)	106 (73.6)

Patients with severe OSA compared to moderate OSA had significantly higher BMI, 43.1 (±11.1) kg/m^2^ vs 38.4 (±8.2) kg/m^2^, respectively (p = 0.013). There was a significant difference in mean STOP-BANG score between severe and moderate OSA, 6.2 (±1.1) vs 5.2 (±1.1) respectively, (p < 0.0001).

There was no significant association between patients’ gender or age and severity of OSA. Higher AHI scores were noted in those ≥50 years old, but this was not significant (p=0.175 and 0.691, respectively).

**Figure 1:**
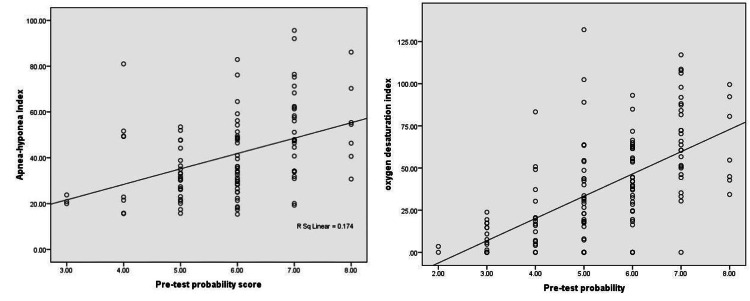
Correlation between AHI/ODI and pre-test probability (STOP-BANG). AHI, apnea-hypopnea index; ODI, oxygen desaturation index

There was a significant positive correlation between AHI, ODI, and STOP-BANG among patients with moderate-to-severe OSA (Figure 1).

**Figure 2:**
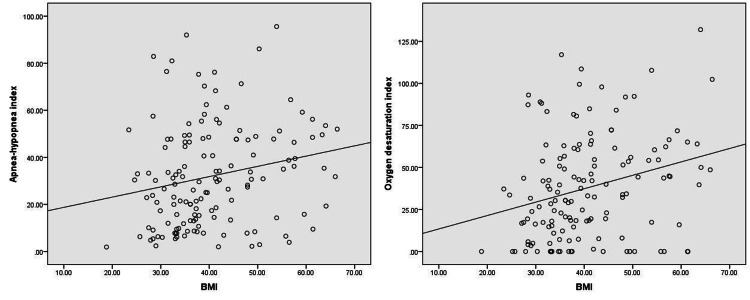
Correlation between AHI/ODI and BMI AHI, apnea-hypopnea index; ODI, oxygen desaturation index; BMI, body mass index

There was a significant positive correlation between AHI, ODI, and BMI of the patients with moderate-to-severe OSA (Figure 2).

**Figure 3:**
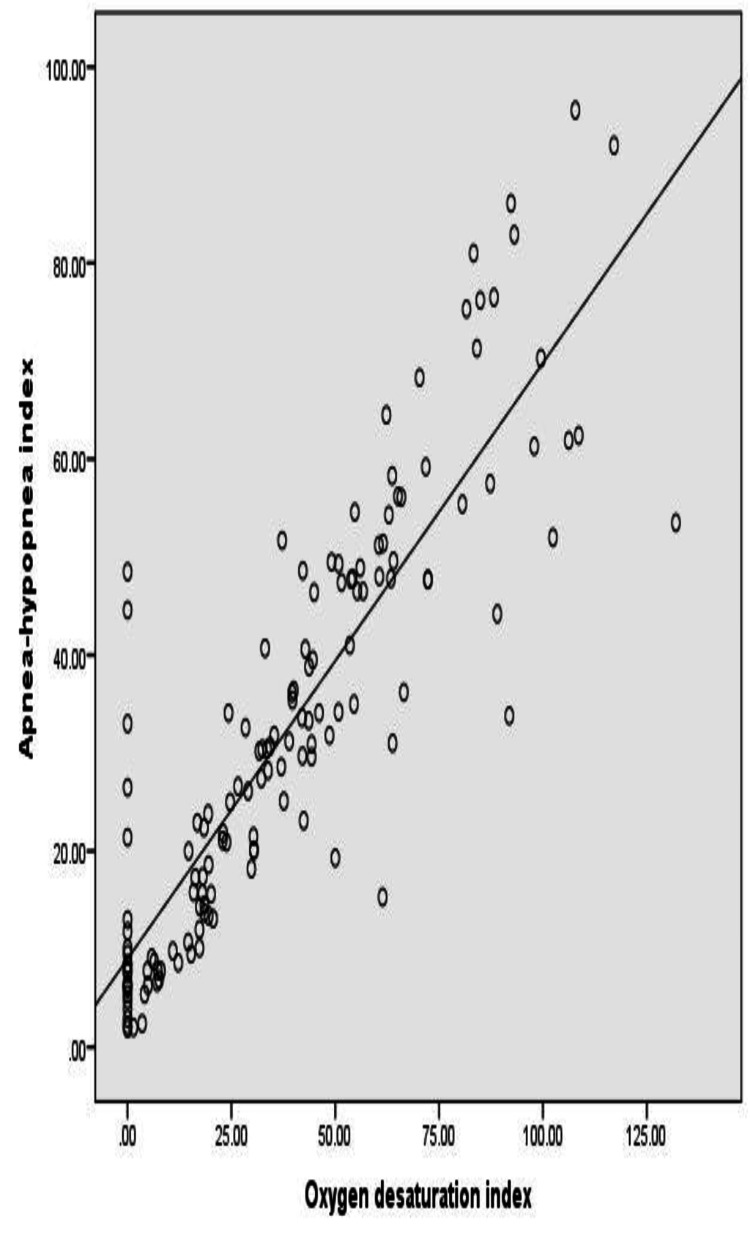
Correlation between AHI and ODI AHI, apnea-hypopnea index; ODI, oxygen desaturation index

There was a strong correlation between the AHI parameters and ODI parameters (Spearman correlation coefficient =0.872; p <0.0001) (Figure 3).

## Discussion

Demographical characteristics are key risk factors in OSA. It has been proven in several studies that obesity, increasing age, and the male gender are risk factors for developing OSA [9,11]]. This was also clearly depicted in our cohort of patients who were predominantly male, above 50 years of age, and obese. The average BMI of our participants was in the range of morbid obesity (BMI > 40kg/m^2^). Differences in the prevalence of OSA among males and females have been clearly documented in the literature [11]. Some of the factors underlying gender disparity include fat distribution, extent of upper airway collapsibility, neurochemical control mechanisms, arousal response, and influence of sex hormones [21]. The enhancing effect of female hormones, especially progesterone, on the tone of pharyngeal muscles has been documented, thereby protecting against upper airway collapse [22]. Thus, reduction of female hormones in the post-menopausal phase explains the almost equal prevalence of OSA in post-menopausal women when compared to men, as the hormonal advantage gets diminished [22]. We, however, noted in this study that although the male population was much higher, the gender disparity was not statistically significant. Since the mean age of our study population was 54±14.8 years, it may be assumed that majority of the females in our cohort could be menopausal, hence the bridge in the prevalence gap.

The high average BMI of patients with moderate-to-severe OSA (>40 kg/m^2^) is worth noting in the current study, as similarly observed in other studies [10,23]. This significant observation in obesity status aligns with findings of a recent study, which showed a persistent increase in the prevalence of overweight and obese individuals among the Ghanaian population from 1998 to 2016 [24]. This increase was predominantly noted in the major cities to be largely driven by changing lifestyles of the public as a result of urbanization and improved middle class status [24]. Rising rates of obesity is of public health concern due to the potential increase in the prevalence of OSA among other obesity-related diseases. The ripple effect from this trend is an escalation in multiple medical comorbidities and invariably healthcare costs in a resource-limited setting as ours. The strong correlation observed between BMI and AHI parameters in our study (r = 0.252; p-value = 0.003) speaks to this concerning link between OSA and obesity. A study by Peppard et al. provided evidence of drop in OSA severity with reduction in obesity [10]. In their report, there was a 26% reduction in AHI values when body weight dropped by 10% [10]. Obesity aggravates fat build-up in the tissues surrounding the upper airway, which leads to increased risk of a narrower pharyngeal airway lumen and a higher risk of collapsibility during sleep [14]. The relationship between OSA and obesity has been shown to be bidirectional, as the former could itself contribute to increased weight gain/obesity influenced by reduced physical activity and increased appetite. This then establishes a vicious cycle between these two independent adverse health risk factors.

OSA is known to be associated with multiple comorbidities, and notable among them are endocrine and metabolic, cardiovascular, and cerebrovascular diseases [17,25]. We similarly report comorbidities in around half of the patients diagnosed with OSA, as in the study by Pinto et al. [25], but we suspect that the high prevalence could have been influenced by the hospital-based sampling. Hypertension, hyperlipidemia, and diabetes were similarly the most commonly reported comorbidities in our cohort [17,25]. The current study, however, found no significant association between AHI scores and patients’ comorbidities, contrasting findings of a previous large epidemiological study [26]. This may be attributed to the retrospective study design with gaps in recorded comorbidities, as well as the limited list of comorbidities provided on the sleep recorded form. The causal link between OSA and development of comorbidities has been controversial as hypertension and obesity are by themselves independent risk factors for adverse cardiovascular, cerebrovascular, and metabolic disorders, and therefore the association may not be etiological [27]. In Ghana, cardiovascular/cerebrovascular disease burden has been shown to be on the rise and the leading cause of mortality [28]. Although OSA is certainly a significant CVD risk factor, its role in the rising burden of cardiovascular disease in our population needs to be unraveled.

The significant linear correlation between AHI/ODI values and the STOP-BANG scores gives credence to the high sensitivity of STOP-BANG sleep questionnaire in identifying patients with moderate-to-severe risk of OSA. The ease of application of STOP-BANG in a busy clinical setting is an advantage over other sleep questionnaires. Interestingly, around 74% of all patients referred for sleep test were identified as high-risk for OSA based on STOP-BANG scores, and around 72% of all referrals were objectively diagnosed as having moderate-to-severe OSA based on AHI categorization. The observed correlation portrays the increasing awareness of OSA among clinicians in Ghana as well as the use of pre-test sleep questionnaires, hence the appropriate referral for sleep testing. The setback is the scarcity of diagnostic tools in the country currently.

Although overnight oximetry, with the generation of ODI scores, has been one of the validated screening tools in OSA, which helps identify patients at high risk of OSA, it has been shown to have similar diagnostic potential as AHI values in patients with severe OSA [29]. The current study finding of a strong linear correlation between AHI and ODI parameters agrees with the above assertion regarding its diagnostic potential. Overnight oximetry can be measured using less expensive portable devices such as ApneaLink and could be valuable in mitigating the lack of OSA diagnostic resources in middle- to low-income countries such as ours [30].

Findings from this first in-country study reveal the growing prevalence of OSA, which was previously believed to be an almost non-existent diagnosis in our clinical practice. The study also established the significant association with obesity, known to be a surging menace in Ghana. We anticipate that these observations will serve as an alert to health policymakers to drive health education campaigns. To better estimate the burden of OSA in our society, future prospective epidemiological studies are required to define the true relationship between OSA and medical comorbidities, particularly cardiovascular diseases.

Study limitations

This was a retrospective study that relied on extraction of manually filed documents. The challenge of missing data and ineligible documentation was real, especially with regard to listing comorbidities. These gaps encountered could have affected the results of the study.

## Conclusions

The absence of objective testing in our setting erroneously gives an impression that OSA is less prevalent and hence not usually a considered differential diagnoses. The study shows that overnight oximetry, a less expensive diagnostic aid can be deployed to improve OSA diagnosis in regional health facilities in the country. The above findings have clearly shown that obesity, a traditional risk factor of OSA may be prevalent in our population and could potentially drive increase in OSA cases.
